# The Food-Specific Serum IgG Reactivity in Major Depressive Disorder Patients, Irritable Bowel Syndrome Patients and Healthy Controls

**DOI:** 10.3390/nu10050548

**Published:** 2018-04-28

**Authors:** Hanna Karakula-Juchnowicz, Mirosława Gałęcka, Joanna Rog, Anna Bartnicka, Zuzanna Łukaszewicz, Pawel Krukow, Justyna Morylowska-Topolska, Karolina Skonieczna-Zydecka, Tomasz Krajka, Kamil Jonak, Dariusz Juchnowicz

**Affiliations:** 1 1st Department of Psychiatry, Psychotherapy and Early Intervention Medical University of Lublin, Gluska Street 1, 20-439 Lublin, Poland; jonak.kamil@gmail.com; 2 Department of Clinical Neuropsychiatry Medical University of Lublin, Gluska Street 1, 20-439 Lublin, Poland; pawelkrukow@umlub.pl (P.K.); justynamorylowska@op.pl (J.M.-T.); 3 Institute of Microecology, Sielska Street 10, 60-129 Poznan, Poland; drgalecka@instytut-mikroekologii.pl (M.G.); anna.bartnicka@instytut-mikroekologii.pl (A.B.); dietetyk@instytut-mikroekologii.pl (Z.L.); 4 Department of Biochemistry and Human Nutrition, Pomeranian Medical University in Szczecin, Broniewskiego Street 24, 71-460 Szczecin, Poland, karzyd@pum.edu.pl; 5 Faculty of Mechanical Engineering, Department of Mathematics, Lublin University of Technology, Nadbystrzycka Street 36, 20-618 Lublin, Poland; t.krajka@pollub.pl; 6 Department of Biomedical Engineering, Lublin University of Technology, Nadbystrzycka Street 38D, 20-618 Lublin, Poland; 7 Department of Psychiatric Nursing Medical University of Lublin, Szkolna Street 18, 20-124 Lublin, Poland; juchnowiczdariusz@wp.pl

**Keywords:** major depressive disorder, irritable bowel syndrome, immunoglobulin G antibody, food antigen, low-grade inflammation, gut-brain axis, food hypersensitivity, food allergy, intestinal permeability

## Abstract

There is an increasing amount of evidence which links the pathogenesis of irritable bowel syndrome (IBS) with food IgG hyperreactivity. Some authors have suggested that food IgG hyperreactivity could be also involved in the pathophysiology of major depressive disorder (MDD). The aim of this study was to compare levels of serum IgG against 39 selected food antigens between three groups of participants: patients with MDD (MDD group), patients with IBS (IBS group) and healthy controls (HC group). The study included 65 participants (22 in the MDD group, 22 in the IBS group and 21 in the HC group). Serum IgG levels were examined using enzyme-linked immunosorbent assay (ELISA). Medical records, clinical data and laboratory results were collected for the analysis. IgG food hyperreactivity (interpreted as an average of levels of IgG antibodies above 7.5 µg/mL) was detected in 28 (43%) participants, including 14 (64%) from the MDD group, ten (46%) from the IBS group and four (19%) from the HC group. We found differences between extreme IgG levels in MDD versus HC groups and in IBS versus HC groups. Patients with MDD had significantly higher serum levels of total IgG antibodies and IgG against celery, garlic and gluten compared with healthy controls. The MDD group also had higher serum IgG levels against gluten compared with the IBS group. Our results suggest dissimilarity in immune responses against food proteins between the examined groups, with the highest immunoreactivity in the MDD group. Further studies are needed to repeat and confirm these results in bigger cohorts and also examine clinical utility of IgG-based elimination diet in patients with MDD and IBS.

## 1. Introduction

Depression is an etiologically and clinically heterogeneous psychiatric disorder [1] which affects more than 300 million people worldwide [2]. Its development is connected with both genetic determinants and environmental factors [3,4]. In 1991, Smith formulated the macrophage theory of depression which suggested that mechanisms involved in the pathogenesis of the disease are macrophage activation and excessive secretion of pro-inflammatory cytokines. Pro-inflammatory cytokines, due to their ability to penetrate the blood–brain barrier (BBB), may influence the metabolism and secretion of neurotransmitters which, consequently, leads to dysregulation of Central Nervous System (CNS) homeostasis [5]. There is a vast body of evidence in support of this theory: (1) the imbalance of pro-inflammatory and anti-inflammatory mediators is observed before the onset of the illness [6]; (2) significantly higher levels of pro-inflammatory cytokines are observed in the acute phase of depression [7]; (3) higher levels of inflammation markers are linked to a higher risk of recurrence of the next depressive episode [8]; (4) higher concentrations of markers of inflammation are connected with progression of the illness [9]; (5) a wide range of anti-inflammatory agents have been successfully tested in patients suffering from major depressive disorder (MDD) [10].

**Figure 1:**
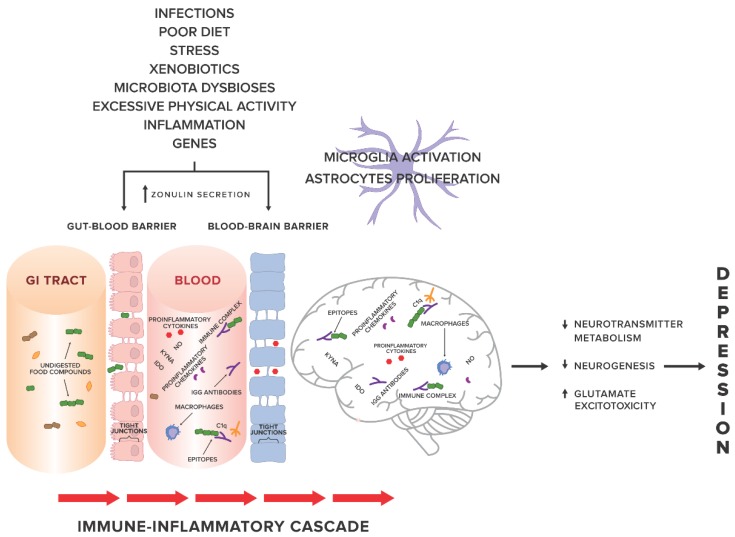
The gut-immune-inflammatory-brain model for Major Depressive Disorder associated with food IgG hyperreactivity. According to the hypothesis proposed in our previous work [19], we present a possible mechanism underlying the MDD development, suggesting that the interplay between genetic and environmental factors may lead to disruption of tight junctions, the loss of their integrity and both gut and BBB permeability. Undigested food compounds, which would normally break down in the gut, translocate into the blood circulation, and trough epitopes combine with food IgG antibodies to form immune complexes. This, in turn, provokes an abnormal response and triggers immune-inflammatory cascade. Uncontrolled release of the proinflammatory mediators may contribute to low-grade systemic inflammation and low-grade neuroinflammation, which, via pathological processes in CNS, i.e., changes in neurotransmitter metabolism, neurogenesis, glutamate excitotoxicity, may in consequence induce and then maintain and prolong depression. Abbreviations: GI tract, gastrointestinal tract; KYNA, kynurenic acid; NO, nitrogen oxide; IDO, indoleamine 2,3-dioxygenase; C1q, complement component 1q.

Factors related to systemic inflammation include i.a. excessive stress, environmental pollution, stimulants (cigarettes, alcohol), excessive body weight, poor diet [11] and leaky gut syndrome [12,13]. An increasing number of studies show a probable relationship between systemic inflammation and gut permeability in such conditions as: coeliac disease [14], autoimmune hepatitis [15], Parkinson’s disease [16], autism spectrum disorders [17,18]. In 2017, Karakula-Juchnowicz et al. suggested that IgG food hypersensitivity could lead to systemic inflammation and be a trigger factor for the development of MDD. Proteins that occur in food and protein-derived compounds may modulate the immune response of the body. Increased gut permeability may lead to translocation of food-borne components into blood circulation, resulting in an abnormal immune response and increased levels of circulating pro-inflammatory cytokines, and thus, the development or maintenance of MDD. Elimination of trigger foods may inhibit the pathological response of the immune system and restore the pro/anti-inflammatory balance of the body [19] (see Figure 1 for an overview).

There is growing evidence that one of the most visible manifestations of gut permeability is irritable bowel syndrome (IBS) which affects 10–13% people around the world [20]. What is more, it is documented that individuals with IBS present low-grade intestinal inflammatory process [21], which is not always connected with a history of a gastrointestinal infection [22]. Irritable bowel syndrome, the most common group of gastrointestinal symptoms characterized by diarrhoea, constipation and bloating, can affect quality of life in those patients [23]. Abdominal pain or discomfort associated with IBS is relieved by defecation. Subtypes of IBS are distinguished based on the manifested symptoms: IBS with constipation (IBS-C), IBS with diarrhoea (IBS-D), mixed IBS (IBS-M), unsubtyped IBS [24].

The etiology of IBS has not been fully elucidated. However, some determinants known as gut-permeability-inducers (e.g., microbiota alternation and diet), have been suggested to play a role in pathophysiology of IBS [25]. Due to the characteristics of IBS (no organic changes), the treatment is based on changes of lifestyle, dietary interventions, counselling, psychological therapies, and dealing with symptoms [26]. Pharmacological therapies that target IBS symptoms include: antispasmodic medication with laxative or anti-diarrhoeal therapies; antibiotics, probiotics, bulking agents, stool softeners or stimulant laxatives [27,28]. Elimination diets are key to reducing IBS symptoms like bloating, gas and pain. The most recommended one is a low FODMAP diet (eliminating fermentable oligosaccharides, disaccharides, monosaccharides and polyols) [29,30] which is followed by a gluten-free diet according to non-celiac gluten sensitivity (NCGS) [31,32], a lactose-free diet [33] or a diet based on measurements of individual levels of IgG antibodies to food antigens in patients [34]. The efficacy of a low FODMAP diet is around 75% in patients with visceral hypersensitivity [35], but on the other hand, a low FODMAP diet might alter gut microbiota. FODMAPs belong to foods that have prebiotic functions, therefore their restriction may lead to reduction in beneficial bacteria in faeces (*Bifidobacteria*) [36].

An elimination diet based on specific IgG antibodies against food has been shown to improve symptoms in patients with IBS [27,37,38]. In 2006, Monsbakken suggested that food hyperreactivity can affect up to 70% of IBS patients [39] and the number of studies confirming this finding is still growing [40,41].

Given that there is:

(1) increasing evidence linking IBS and food IgG hyperreactivity, and
(2) a suggested relationship between food IgG reactivity and MDD.

We decided to compare IgG reactions to food proteins in the serum of patients with IBS, MDD and healthy controls.

## 2. Material and Methods

### 2.1. Participants

Sixty five subjects aged 18–60 years participated in the study, including: 22 outpatients with a *DSM-5* diagnosis of major depressive disorder (MDD) [42], 22 outpatients who met Rome III Criteria [24] for the diagnosis of irritable bowel syndrome (IBS) and 21 healthy controls (HC) with no current or past history of IBS or psychiatric disorders. The mean age and sex ratio were matched across the three groups.

Exclusion criteria were as follows: (1) Body Mass Index (BMI) below 18.5 kg/m^2^ or above 30 kg/m^2^ (due to the possibility of either excessive or insufficient exposure to antigens, abnormal gut permeability [43,44,45] and changes in the gut microbiota among underweight and overweight subjects [45,46,47]); (2) prior or current medical history of organic brain dysfunctions; (3) a lifetime IBS diagnosis in the MDD group; (4) a lifetime MDD diagnosis in the IBS group (5) meeting criteria for substance abuse/dependence or mental retardation; (6) current use of anti-inflammatory or anti-allergic medications or antibiotics; (7) any inflammatory, oncological or systemic immune disease, diabetes mellitus, infectious diseases; (8) following any types of specific diets; (9) pregnancy or lactation.

The study was conducted in accordance with the Declaration of Helsinki, and the protocol was approved by the Ethics Committee of Medical University of Lublin (the project identification code: KE-0254/127/2016). After description of the study, a written informed consent was obtained from every subject.

### 2.2. Concentration of Specific IgG Antibodies

The fasting sera were examined for the concentration of specific IgG against 39 selected foods using enzyme-linked immunosorbent assay (ELISA), according to the manufacturer’s recommendations (ImuPro test; R-Biopharm, Darmstadt, Germany), which was described previously [48]. Standardized calibration curve for IgG was performed according to 1st WHO IRP 67/86 for human IgG. Quantitative measurements are shown in µg/mL. All values above 7.5 µg/mL were considered as a positive reaction to a certain food. The full list of food antigens tested includes: vegetables (broccoli, carrot, cucumber, sweet pepper, red cabbage, tomato, celery, soya beans); fruits (pineapple, watermelon, cherry); spices (horseradish, garlic, mustard seed); meat (pork, beef, chicken); eggs (chicken egg); cereals with gluten (gluten, barley, oat, wheat, spelt, rye); seeds and nuts (poppy seed, almond, hazelnut, peanut, pistachio, linseed, sunflower seed); fish & seafood (crayfish); milk products (cow’s: milk, sour-milk products, rennet cheese, goat’s: milk and cheese, sheep’s: milk and cheese); sugar products (honey) and yeast (bakery yeast).

### 2.3. Statistical Analysis

Earlier studies demonstrated the skewed distributions of data on IgG antibodies concentrations [49]. In view of the possibility of weakness of results obtained from classical statistical analysis and changes the reliability of analysis after a log-transformation we decided to use the ex-Gaussian statistics which is more advanced and could detect an important dissimilarity in groups with extreme results of IgG plasma levels.

The ex-Gaussian modelling of raw biological data was conducted to illustrate the specificity of quantitative results in a way that best reflects the distribution of the parameters studied. The ex-Gaussian statistics were used to compare the unprocessed results we obtained for the groups, without the need to remove the selected results or to convert the results artificially in order to adjust them to Gaussian distribution, but to take into account their exponential specificity.

The use of the ex-Gaussian statistics makes it possible to estimate three independent parameters: mu (μ) representing the mean of the normal component, reflecting the average IgG results in each group, sigma (σ) corresponding to the symmetrical standard deviation of the normal component, showing the variability of raw results, and tau (τ), containing the exponential part of the distribution, displaying the extremes in IgG results. Comparisons of mu and tau between three studied groups will show factual differences in two separated aspects of IgG outcomes: averaged, most frequently occurring results, and the comparison of tau will indicate which group has the highest number of individuals with clinically high IgG titers.

**Figure 2:**
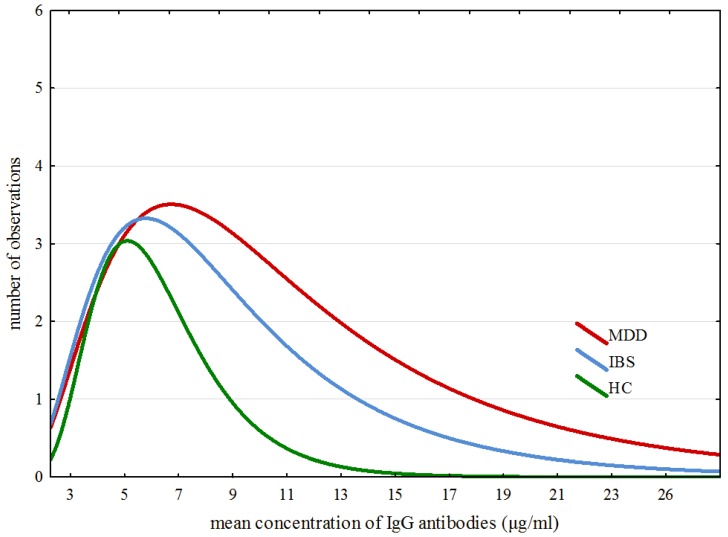
Specific traits of distribution of averaged results across all groups.

The modelling of ex-Gaussian parameters was performed with the MATLAB toolbox “DISTRIB”. Data pre-processing allowing correct export of the results to the MATLAB software was proceeded with an individually customized Excel macro. All further operations were conducted according to Lacouture and Cousineau [50] recommendations for applying ex-Gaussian modelling to experimental data. Figure 2 confirms that the data obtained for all IgG results in all three groups match closely to the specificity of the ex-Gaussian distribution.

Due to the skewed distribution of IgG data, the comparison of obtained ex-Gaussian parameters (μ, σ and τ) for three groups was conducted with the omnibus Kruskal–Wallis rank-based nonparametric H test for comparison of more than two groups. In the case of results confirming the significant between-group difference, *post-hoc* analysis was performed with the median test, an extension of Kruskal–Wallis test enabling the comparison of all groups in pairs. This solution has been chosen instead of Mann–Whitney statistics as a *post-hoc* test, because the ranks that the pair-wise Mann–Whitney test use are not the ranks used by the Kruskal–Wallis test. Relinquishing of the Mann-Whitney test as a *post-hoc* analysis method will also reduce the risk of type I error occurrence [51].

The further statistical exploration was focused on establishing the between-group differences regarding the individual IgG results. This step of the statistical analysis was dependent on the result of the comparison concerning the ex-Gaussian parameters. When the comparison showed a significant difference between two groups, the Mann–Whitney test was used. When the difference concerned three groups, the Kruskal–Wallis test was applied, similarly as with the ex-Gaussian parameters.

All statistical analyses were performed using STATISTICA 12 (TIBCO Software Inc., CA, USA) for Windows and MATLAB software (version: R2017a, Mathworks Inc., Natick, MA, USA).

## 3. Results

### 3.1. Characteristics of the Examined Groups

General characteristics of the participants are shown in Table 1. There were no differences in age or alcohol intake between the examined groups. The MDD group had higher BMI and lower levels of physical activity compared with the IBS group. The median of coffee intake (cups/day) was higher in MDD (two cups of coffee a day) compared with IBS and HC group (0 cup of coffee a day). Interestingly, both MDD and the IBS group had more severe gastric complaints (measured during the week prior to the examination by the scale which subjectively assessed frequency and severity of symptoms; the sum of complaints ranged from 0 to 10) compared with the HC group.

Duration of illness ranged from 0.5 to 22 years across the MDD group and from 3 to 36 years across the IBS group. Three (13.63%) of the MDD patients experienced their first episode of depression, 18 (86.37%) patients had a recurrent episode of depression (the number of episodes ranging from two to 11 episodes). Eleven (50%) from MDD patients received selective serotonin reuptake inhibitors (SSRIs), four (18.18%) patients were taking Venlafaxine, five (22.73%) patients were treated with Trazodone and two (9.09%) with Mianserine. Six (31.82%) from the IBS patients had IBS with constipation, four (18.18%) IBS with diarrhoea, five (22.73%) mixed IBS and three (13.64%) unspecific IBS.

Twenty-nine (44.62%) of the patients reported using a diet in the past. Five (7.70%) patients had tried a diet with caloric restriction, including two (9.09%), two (9.09%) and one (4.76%) participants from MDD, IBS and HC group, respectively. Among sixteen (24.62%) patients who had been following an elimination diet, there had been six (27.27%) patients from the MDD group, six (27.27%) from the IBS group and four (19.05%) from HC group (see Supplementary Materials Table S1). At the moment of the examination, none of the patients were on a diet, which was one of the exclusion criteria.

### 3.2. Seroreactivity to Food Antigens in the Examined Groups

IgG food hyperreactivity (interpreted as an average of levels of IgG antibodies above 7.5 µg/mL) was detected in 28 (43%) participants: 14 (64%) from the MDD group, ten (46%) from the IBS group and four (19%) from the HC group.

Due to substantially skewed data with increased exponential part of distribution, we decided to examine the differences in the ex-Gaussian parameters (which include differences of the normal component— σ and the extremes— τ) of total IgG serum levels between the three groups.

Figure 2 shows specific traits of distribution of averaged results across all three groups. Detailed analysis of ex-Gaussian distribution of total IgG values suggests a dissimilarity in immune responses between participants from the examined groups. The outliers of IgG levels are shaped from approximately 19–28, from approximately 14–20 and from approximately 9–13 in the MDD, IBS and HC groups, respectively.

As shown in Table 2, there were no significant differences in μ and σ parameters between the three groups. However, statistically significant differences were found in τ parameter between the examined groups. The *post-hoc* analysis revealed differences between MDD and HC groups and between IBS and HC groups.

The comparison of serum IgG levels against various food proteins between the examined groups analysed by Kruskal–Wallis non-parametric ANOVA and the results after *post-hoc* test are shown in Table 3. We present only statistically significant results. A comparison of IgG antibodies against all analysed food proteins was summarized in Supplementary Materials (see Tables S2 and S3).

There was a difference in total serum IgG levels between MDD and HC groups. Patients suffering from MDD had significantly higher serum levels of total examined IgG antibodies. An analysis of individual IgG levels revealed that patients with MDD had significantly higher IgG levels against broccoli, celery, horseradish, garlic, gluten, wheat, rye and milk products compared with HC group. The MDD group also had statistically higher serum IgG levels against gluten and sunflower seed compared with IBS patients. After *post-hoc* analysis, statistical significance was achieved only by results in differences of serum concentrations of IgG against celery, garlic and gluten between MDD and HC groups and against gluten between MDD and the IBS groups.

We find no correlations between gender, age, BMI, number of cigarettes and cups of coffee per day, intake of alcohol, physical activity, duration of illness and serum total IgG levels across the examined groups. Similarly, correlations between gastric complaints and serum IgG levels were not found in IBS and HC groups. However, there was a positive correlation between total IgG level and gastric complaints in the MDD group (*p* < 0.05; *R* = 0.66).

We performed another statistical analysis on the group of IBS patients divided into two subgroups, i.e., with diarrhoea (IBS-D) and non-diarrhoea (IBS-ND). We did not find any differences either in the total IgG levels (*p* > 0.05) or in the levels of specific IgG antibodies (*p* > 0.05) between the IBS-D and IBS-ND patients. Focusing on differences between IBS subtypes seems to be a promising avenue for future research on bigger groups of patients suffering from IBS. As for our study, due to unequal numbers of patients in 4 groups resulting from the division of the IBS group, multiple-group analysis could not be conducted.

## 4. Discussion

There are numerous studies which confirm IgG hyperreactivity to food antigens in patients with IBS [27,38,52,53,54,55,56,57,58,59].

Due to this substantial amount of evidence and an unclear association between MDD and IgG hyperreactivity, we decided to compare levels of IgG antibodies against food between IBS, MDD patients and healthy controls.

The results of our study indicate differences between the examined groups in the proportion of people whose total IgG values exceeded the cut off level (>7.5 µg/mL). Unusually, most cases of hyperreactivity were found in MDD (64%) group and the least number in HC (19%) group. As revealed by the *post-hoc* comparisons of the paired groups, total IgG in the examined groups showed differences between MDD patients and healthy controls, no such dissimilarity was found between the IBS group and HC group. It was the application of ex-Gaussian modelling (due to substantially skewed data with increased exponential part of distribution) that revealed differences in tau (τ) (which displays the extremes in IgG results) between MDD and HC groups and also between the IBS and HC groups.

Further analysis demonstrated the presence of many distinctions in individual levels of IgG depending on the type of illness. There were differences in levels of IgG against celery, garlic and gluten between MDD and HC groups and levels of IgG against gluten between the IBS and MDD groups.

Our results indicate a dissimilarity in the immune response to food antigens among the three studied groups, surprisingly, with the highest immunoreactivity in patients suffering from MDD compared with healthy controls. A possible mechanism of the phenomenon remains obscure.

On the one hand, a possible cause of the hyperreactivity among patients with MDD may be disruption of gut-microbiome-brain axis, which is related to low-grade inflammation occurring peripherally and in the CNS [19]. It is suggested that altered gut microbiota could be responsible for increased gut permeability.

Evidence from model studies confirms that intragastric administration of *Clostridium butyricum* contributes to improvements in mucosa and BBB integrity and inhibition of neuroinflammation processes [60]. What is more, one recent study on patients with mood disorders revealed that the disruption of gut barrier integrity could be a result of gut dysbiosis. Based on the analysis of fecal microbiota, researchers demonstrated that patients with MDD and anxiety were characterized by over-representation of genes involved in LPS biosynthesis and deleterious metabolism of mood neurotransmitter pathways. The patients with mood disorders had also higher levels of plasma LPS, zonulin and fatty-acid binding protein 2 (FABP2) which reflected gut permeability [61]. However, it is still poorly understood whether intestinal barrier dysfunction is a cause or a consequence of MDD or it exists merely in some group of patients regardless of the disease.

On the other hand, there is some evidence that due to gut-brain signaling, inflammation of CNS could lead to secondary disruptions of the digestive tract. More and more studies show that neuroinflammation is a cause of chronic mucosal damage in the gut and hyperreactivity of enteric glial cells. The intestinal barrier disruptions manifest themselves by long-term neurobehavioral changes [62,63]. Post-mortem studies demonstrated that neuroinflammation was involved in pathophysiology of depression [64,65].

A scenario proposed by Karakula-Juchnowicz et al. that links the inflammatory theory of depression with IgG food hypersensitivity and leaky gut syndrome assumes that loss of integrity of the tight-junction barrier could be caused by food antigens [19]. Overproduction of zonulin triggered, for example, by gliadin through activation of the epidermal growth factor receptor and protease-activated receptor causes loosening of the tight junction barrier and an increase in permeability of the gut wall. Afterwards, intestinal permeability allows food-derived compounds cross into bloodstream and activate the immune response which is involved in overproduction of proinflammatory cytokines, their transport across BBB and, consequently, the presence of clinical manifestation of depression [19]. There is a substantial body of evidence that there is a link between food-derived antigens and other psychiatric disorders with the suggested role of inflammation in their pathogenesis [66]. Okusaga et al. revealed a possible relationship between non-celiac gluten sensitivity dependent on IgG antibodies and inflammatory pathways. In the mentioned study, schizophrenic patients with elevated anti-gliadin IgG had increased serum kynurenine levels and kynurenine to tryptophan ratio compared with patients without increased anti-gliadin IgG. Tryptophan to kynurenine conversion is linked with inflammation and raised cortisol levels [67]. Another study found that anti-casein IgG antibodies are linked with type I bipolar disorder, psychotic symptoms and mania severity [68].

So far, the number of studies exploring seroreactivity to food antigens in patients with MDD has been insufficient. Only one observation has been made and its results remain inconclusive. The paper in question reports no differences in mean IgG concentrations between MDD patients and healthy persons. The researchers observed lower levels of IgG against dietary proteins in patients with MDD who had high exposure to milk proteins compared with the control group with high exposure. Surprisingly, the authors found lower levels of tumour necrosis factor-alpha (TNF-alpha) and higher levels of cortisol in sera of the patients relative to the control subjects [69]. TNF-alpha and interleukin 6 (IL-6) are considered to be well-known inflammatory factors involved in pathophysiology of depression, as supported by a meta-analysis done by Köhler et al. in 2017 [70]. Also, the results of the studies on anti-inflammatory effects of cortisol [71] have not been confirmed in MDD patients where excess cortisol secretion was associated with higher levels of proinflammatory cytokines [72]. These findings confirmed the notion that glucocorticoid resistance, cortisol hypersecretion and increased inflammation are indeed coexistent and related biological abnormalities [73]. An interesting observation made by Rudzki et al. [69] is the link between TNF-alpha concentration and total food IgG level, indicating a potential relationship between IgG reactivity to dietary proteins and low-grade inflammation. Some explanation for the discrepant results obtained by Rudzki et al. [69] may be provided by findings from the study by Lamers at al. [74] pointing to a differential role of HPA-axis function, inflammation and metabolic syndrome in melancholic versus atypical depression. The proportion of 50% of patients with melancholic depression versus 11.76% of patients with atypical depression in the study by Rudzki et al. [69] could have had a substantial influence on a lack of differences in IgG levels between the patients and the healthy controls. A relatively small size of the group of patients and statistical analysis conducted by means of classical methods may also have affected the validity of the observations.

Our results regarding differences between IBS and HC are partly consistent with findings from other studies and indicated that serum IgG antibody levels of some common foods are abnormally elevated in IBS patients [75,76]. We did not find differences in total IgG levels between IBS and HC groups until we applied ex-Gaussian modelling which allowed us find differences in the extreme results (τ) of IgG levels between these two groups. Based on elevated IgG above the cut-off point we detected dissimilarity between IBS (46%) and HC (19%). However, we did not find differences in individual levels of IgG.

This lack of consistency with findings from other studies could be a consequence of a relatively small size of the group. On the other hand, this may be explained by attempts of IBS patients to cut down on foods aggravating their symptoms such as bloating or constipation. Taking into account that gastrointestinal complaints are the core of IBS [77], patients suffering from this disorder might intuitively cut down on the food products that, in their opinion, worsen the symptoms [39,78], yet without calling this practice “following a diet”. From a clinical perspective, such behaviour is not tantamount to an elimination diet, which was an exclusion criterion from our study. However, it might lead to decreased exposure to a specific food antigen, possibly affecting IgG levels. For this reason, further research aimed at finding differences in food IgG levels between the groups studied should determine food intake and eating frequency in order to evaluate exposure to the food products studied. It is worth mentioning that an elimination diet is a method used to reduce IgG antibodies in the case of high concentrations of IgG against food proteins [78]. On the other hand, results of studies concentrated on the relationship between food-IgG exposure and dietary habits are contradictory [69,79], so research aimed at assessing differences in IgG antibodies against food, especially food with high FODMAP concentration levels (e.g., garlic, gluten, broccoli), between healthy controls and IBS patients should strictly determine the time between the discontinuation of the diet and IgG examination.

A positive response to a diet based on IgG antibodies against food has been reported in several different diseases, such as migraine [49,80], obesity [81], Crohn disease [82]. A common thread of all above cases is inflammation. Recent data provide evidence that inflammatory processes are considered to be an important factor for the development of both depression and IBS [12,83,84]. To our knowledge, there are no randomized control trials supporting potential utility of an elimination diet in patients with MDD, however, it has been shown that an elimination diet based on specific IgG antibodies against food may alleviate symptoms in patients suffering from IBS [27,37,38,39].

In a double blind, randomized, controlled, parallel study, Atkinson et al. [27] showed significant reduction in severity of symptoms in patients with IBS due to an elimination diet based on a IgG test compared to a sham diet (not based on increased quantities of IgG-antibodies). The quality of life also improved after following an IgG-based diet. The authors calculated that three patients out of four should be treated with this approach. After reintroduction of the respective foods the researchers observed the reverse response in the patients. Those findings are consistent with a study by Drisko et al. [38]. They also show a significant improvement of symptoms in patients with IBS. The researchers demonstrated increased titres of specific IgG antibodies for selected food in this group of patients. After 6 months on a diet without products with increased IgG concentration, patients with IBS demonstrated improvement in stool frequency, pain relief and the quality of life.

In turn, Zuo et al. [52] analysed patients with IBS and FD (functional dyspepsia) in comparison to the control group in terms of specific IgG, IgE antibody and total IgE antibody titres. They demonstrated that IBS patients had significantly higher titres of IgG antibody to crab, egg, shrimp, soybean and wheat than controls. FD patients had significantly higher titres of IgG antibody to egg and soybean than controls. Interestingly, there was no correlation in percent of patients with positive specific IgE antibodies in the three groups. There were no significant differences between IBS patients, FD patients and controls in the serum total IgE antibody levels. Guo et al. [57] evaluated the benefit of an IgG-based diet in patients with IBS with diarrhoea form (IBS-D) of the disease. The phase of 12-week elimination of food products with increased IgG resulted in improvement in IBS-D participants compared with the baseline. The researchers showed amelioration in abdominal pain (bloating level and frequency), diarrhoea frequency, abdominal distension, stool shape, general feelings of distress and total symptom score.

An interesting study was conducted by Aydinlar et al. [56]. In this double-blind, randomized, controlled, cross-over clinical trial individuals suffering from both migraine and IBS could take advantage of IgG-based elimination diet. An individual diet approach may effectively alleviate conditions such as migraine attack, maximum attack duration, mean attack duration, maximum attack severity and number of attacks with acute medication, as well as abdominal symptoms: pain-bloating severity, pain-bloating within the last 10 days, and also improvement in quality of life. Nonetheless, the small sample (21 patients) and funding bias of the examination should be pointed out as a weakness of the study.

Despite many results confirming the clinical manifestation of elevated levels of IgG antibodies against food antigens, the diagnosis based on their elimination is controversial. Numerous allergy societies question the usefulness of IgG antibodies tests as a diagnostic method for assessing adverse reactions to food intake [85,86,87,88], nevertheless, some recommend using IgG antibodies tests for research purposes [88]. It is known that IgG4 is an antibody involved in the desensitization of type I allergies (IgE-dependent) [89]. There is some new evidence that patients with IBS my present increased IgG4 titres [76], but this still needs clarification. It is worth mentioning that not all subclasses of IgG are involved in desensitization. Furthermore, they are able to form an immune complex with bounded food antigens. Such complexes are destroyed by pathogenic cells which leads to the release of proinflammatory cytokines. Aljada et al. showed that food intake is able to induce significant inflammatory changes, which has been characterized by a decrease in IkappaBalpha and an increase in NF-kappaB binding, plasma C-reactive protein (CRP), and the expression of IKKalpha, IKKbeta as well as p47 (phox) subunit [90]. This state induces a low-grade inflammation condition which may be aggravating for the body. It is documented that individuals with IBS may present low-grade inflammatory process in the gut mucosa which is not always connected with a history of a gastrointestinal infection [22]. Therefore, testing IgG against food could be one of the novel diagnostic approach to identify triggers leading to inflammation in these conditions, and administration of diet based on concentration of specific IgG may exert a beneficial effect in patients with IBS and depression.

## 5. Conclusions

Multiple lines of evidence increasingly point towards a role of environmental factors and disrupted gut–brain axis function both in many neuropsychiatric disorders, including depression, and in gastrointestinal disorders, including IBS [19,91,92,93]. The wide range of factors involved in the disturbances are considered, including changes in the mucosal immune system, the microbiota dysbioses and amounts of short chain fatty acids dependent on microbiota, exposure to xenobiotics, gut permeability, food IgG antibodies [91,92,93,94,95].

Our findings suggest more common food-specific serum IgG hyperreactivity among patients with IBS and MDD compared with HC, which may be one of the mechanisms leading to the development of immune activation and low-grade inflammation observed in these disorders.

Interestingly, the highest reactivity was observed in the group of MDD patients, the fact that may be of great importance for both theoretical considerations on MDD etiopathogenesis and therapeutic implications for this disorder. There is no causal relationship which could confirm clinical utility of an elimination diet in patients with MDD, however, some evidence suggests reduction of symptoms in patients with IBS who followed an IgG-based elimination diet.

To the best of our knowledge, this is the first paper published worldwide to compare IgG reactivity to food antigens in patients with these two disorders and we are of the opinion that further studies are needed to repeat and confirm these results in bigger cohorts and to examine clinical utility and duration of IgG-based elimination diet in patients with MDD and with IBS.

## Figures and Tables

**Table 1:** Characteristic of studied groups.

	MDD (*n* = 22)	IBS (*n* = 22)	HC (*n* = 21)	Analysis	*Post-hoc* Analysis
**Age**	31.5 (14.5)	38.5 (15.3)	34 (25)	H = 1.36, *p* = 0.506	–
**Gender (% male)**	50	45.4	57.1	χ^2^ = 1.17, *p* = 0.554	–
**BMI**	28.6 (3.2)	22.8 (5.5)	25.8 (5.9)	H = 9.59, *p* = 0.005	MDD > IBS
Physical activity *	1 (0)	2 (0.3)	2 (1.5)	H = 7.77, *p* = 0.021	MDD < IBS
**Number of cigarettes per day**	0 (2.25)	0 (0)	0 (0)	H = 2.45, *p* = 0.294	–
**Number of cups of coffee per day**	2 (2)	0 (0)	0 (2)	H = 22.32, *p* <0.001	MDD > IBS MDD > HC
**Intake of alcohol**	4 (7.5)	4 (1)	3.5 (3.3)	H = 2.11, *p* = 0.348	–
Gastric complaints *	6.5 (6)	5 (5.5)	1 (3)	H = 16.25, *p* <0.001	MDD > HC IBS > HC
**Duration of illness**	5 (8)	3 (11.5)	–	Z = 0.94, *p = 0.35*	–

Values are shown as median (interquartile range); MDD—major depressive disorder; IBS—irritable bowel syndrome; HC—healthy controls; BMI—body mass index; H—H-value; *p*—*p*-value; χ^2^—χ^2^-value; Z—Z-value; * based on 10-point scale.

**Table 2:** Differences in IgG normal part of data distribution (µ), symmetrical standard deviation (σ) and exponential part of IgG levels dispersion (τ).

ex-Gaussian Parameters	MDD *M*	IBS *M*	HC *M*	H (2, 65)	*p*	*Post-hoc* Analysis
µ	1.640	1.820	2.027	1.178	0.554	–
σ	0.001	0.175	0.463	0.883	0.642	–
τ	8.088	5.692	2.616	19.389	0.0001	MDD > HC IBS > HC

*Post-hoc* analysis: MDD-HC: major depressive disorder–healthy controls, *p* < 0.00001; IBS–HC: irritable bowel syndrome–healthy controls, *p* = 0.018; *M*—median; H—Kruskal-Wallis non-parametric ANOVA; *p*—*p*-value.

**Table 3:** Differences in serum IgG levels against tested food proteins.

IgG	G	M	IQR	Min	Max	Kruskal–Wallis H Test for 3 Groups		Post-hoc Analysis	
						H	p	Groups	p
Total IgG	MDD	403.9	365.1	109.68	1075.11	7.90	0.019	MDD > HC	0.004
	IBS	308.6	282.1	108.07	1041.08				
	HC	219.3	70.2	130.20	657.15				
Broccoli	MDD	5.29	3.16	1.52	21.85	6.20	0.045	MDD > HC	0.039
	IBS	4.73	2.72	1.79	10.43				
	HC	3.56	1.04	1.62	6.40				
Celery	MDD	6.15	6.80	1.58	28.20	8.03	0.017 *	MDD > HC	0.019
	IBS	4.53	4.74	1.52	12.43				
	HC	2.87	2.14	1.14	7.32				
Horseradish	MDD	4.08	3.66	1.42	48.10	6.98	0.030	MDD > HC	0.024
	IBS	4.02	2.68	1.51	11.43				
	HC	2.77	0.89	1.08	6.79				
Garlic	MDD	6.33	7.02	1.01	75.64	7.88	0.017 *	MDD > HC	0.015
	IBS	2.92	4.98	0.75	15.83				
	HC	2.49	1.19	0.95	10.77				
Gluten	MDD	16.44	16.10	5.26	112.61	10.37	0.005 *	MDD > HC	0.025
	IBS	8.87	10.43	2.26	117.87				
	HC	11.74	7.40	3.94	43.01			MDD > IBS	0.010
Wheat	MDD	15.26	16.38	4.56	99.42	6.79	0.033	MDD > HC	0.043
	IBS	9.31	8.05	2.28	122.13				
	HC	8.60	5.53	3.28	47.22				
Rye	MDD	11.41	8.42	3.73	108.92	7.23	0.026	MDD > HC	0.032
	IBS	7.38	8.14	2.10	28.52				
	HC	5.59	4.94	2.08	38.05				
Sunflower seed	MDD	6.22	5.84	1.43	46.15	7.07	0.029	MDD > IBS	0.038
	IBS	3.06	2.94	0.91	68.05				
	HC	3.13	2.45	0.74	61.62				
Milk products	MDD	27.57	39.82	1.62	110.57	7.59	0.025	MDD > HC	0.019
	IBS	20.00	42.78	1.24	119.30				
	HC	6.86	5.46	0.96	86.24				

* Differences statistically significant also after *post-hoc* analysis; G—group; *M*—median; IQR—interquartile range; H—H-value; *p*—*p*-value. MDD—major depressive disorder; IBS—irritable bowel syndrome; HC—healthy controls.

## Supplementary Materials

The following are available online at http://www.mdpi.com/2072-6643/10/5/548/s1. Table S1: Types of diets followed by participants in the past. Table S2: Differences in serum IgG levels against tested food proteins, Table S3: Frequency of elevated IgG levels in examined groups.
