# Supplementary material for: The Food-Specific Serum IgG Reactivity in Major Depressive Disorder Patients, Irritable Bowel Syndrome Patients and Healthy Controls
Source: Nutrients. 2018 Apr 28;10(5):548. doi: 10.3390/nu10050548 (PMC5986428; doi:10.3390/nu10050548)
Supplement: Supplementary file 1 [file nutrients-10-00548-s001.zip › supplementary materials/Table 3.docx]

**Table 3.** Differences in serum IgG levels against tested food proteins

| **IgG** | **G** | ***M*** | **IQR** | **Min** | **Max** | **Kruskal-Wallis H Test for 3 groups** | | ***Post-hoc* analysis** | |
| --- | --- | --- | --- | --- | --- | --- | --- | --- | --- |
|  |  |  |  |  |  | **H** | **p** | **groups** | **p** |
| Total IgG | MDD | 403.9 | 365.1 | 109.68 | 1075.11 | 7.90 | 0.019 | MDD > HC | 0.004 |
|  | IBS | 308.6 | 282.1 | 108.07 | 1041.08 |  |  |  |  |
|  | HC | 219.3 | 70.2 | 130.20 | 657.15 |  |  |  |  |
| Broccoli | MDD | 5.29 | 3.16 | 1.52 | 21.85 | 6.20 | 0.045 | MDD > HC | 0.039 |
|  | IBS | 4.73 | 2.72 | 1.79 | 10.43 |  |  |  |  |
|  | HC | 3.56 | 1.04 | 1.62 | 6.40 |  |  |  |  |
| Celery | MDD | 6.15 | 6.80 | 1.58 | 28.20 | 8.03 | 0.017* | MDD > HC | 0.019 |
|  | IBS | 4.53 | 4.74 | 1.52 | 12.43 |  |  |  |  |
|  | HC | 2.87 | 2.14 | 1.14 | 7.32 |  |  |  |  |
| Horseradish | MDD | 4.08 | 3.66 | 1.42 | 48.10 | 6.98 | 0.030 | MDD > HC | 0.024 |
|  | IBS | 4.02 | 2.68 | 1.51 | 11.43 |  |  |  |  |
|  | HC | 2.77 | 0.89 | 1.08 | 6.79 |  |  |  |  |
| Garlic | MDD | 6.33 | 7.02 | 1.01 | 75.64 | 7.88 | 0.017* | MDD > HC | 0.015 |
|  | IBS | 2.92 | 4.98 | 0.75 | 15.83 |  |  |  |  |
|  | HC | 2.49 | 1.19 | 0.95 | 10.77 |  |  |  |  |
| Gluten | MDD | 16.44 | 16.10 | 5.26 | 112.61 | 10.37 | 0.005* | MDD > HC | 0.025 |
|  | IBS | 8.87 | 10.43 | 2.26 | 117.87 |  |  |  |  |
|  | HC | 11.74 | 7.40 | 3.94 | 43.01 |  |  | MDD > IBS | 0.010 |
| Wheat | MDD | 15.26 | 16.38 | 4.56 | 99.42 | 6.79 | 0.033 | MDD > HC | 0.043 |
|  | IBS | 9.31 | 8.05 | 2.28 | 122.13 |  |  |  |  |
|  | HC | 8.60 | 5.53 | 3.28 | 47.22 |  |  |  |  |
| Rye | MDD | 11.41 | 8.42 | 3.73 | 108.92 | 7.23 | 0.026 | MDD > HC | 0.032 |
|  | IBS | 7.38 | 8.14 | 2.10 | 28.52 |  |  |  |  |
|  | HC | 5.59 | 4.94 | 2.08 | 38.05 |  |  |  |  |
| Sunflower seed | MDD | 6.22 | 5.84 | 1.43 | 46.15 | 7.07 | 0.029 | MDD > IBS | 0.038 |
|  | IBS | 3.06 | 2.94 | 0.91 | 68.05 |  |  |  |  |
|  | HC | 3.13 | 2.45 | 0.74 | 61.62 |  |  |  |  |
| Milk products | MDD | 27.57 | 39.82 | 1.62 | 110.57 | 7.59 | 0.025 | MDD > HC | 0.019 |
|  | IBS | 20.00 | 42.78 | 1.24 | 119.30 |  |  |  |  |
|  | HC | 6.86 | 5.46 | 0.96 | 86.24 |  |  |  |  |

*differences statistically significant also after post-hoc analysis
G – group; *M* – median; IQR - interquartile range; H – H-value; p – p-value
