# Supplementary material for: The Food-Specific Serum IgG Reactivity in Major Depressive Disorder Patients, Irritable Bowel Syndrome Patients and Healthy Controls
Source: Nutrients. 2018 Apr 28;10(5):548. doi: 10.3390/nu10050548 (PMC5986428; doi:10.3390/nu10050548)
Supplement: Supplementary file 1 [file nutrients-10-00548-s001.zip › supplementary materials/Table S1.pdf]

Table S1. Types of diets followed by participants in the past

Study group	Type of diet number of subjects (%)			
	No diet	Caloric restricted	Elimination diet	Other
MDD	13 (59.09)	2 (9.09)	6 (27.27)	1 (4.55)
IBS	8 (36.37)	2 (9.09)	6 (27.27)	6 (27.27)
HC	15 (71.43)	1 (4.76)	4 (19.05)	1 (4.76)
