# Supplementary material for: The Food-Specific Serum IgG Reactivity in Major Depressive Disorder Patients, Irritable Bowel Syndrome Patients and Healthy Controls
Source: Nutrients. 2018 Apr 28;10(5):548. doi: 10.3390/nu10050548 (PMC5986428; doi:10.3390/nu10050548)
Supplement: Supplementary file 1 [file nutrients-10-00548-s001.zip › supplementary materials/Table S2.pdf]

Table S2. Differences in serum IgG levels against tested food proteins

IgG	G	*M*	IQR	Min	Max	Kruskal-Wallis H Test for 3 groups		*Post-hoc* analysis	
						H	p	groups	p
Total IgG	MDD	403.9	365.10	109.68	1075.11	7.90	0.019	MDD > HC	0,004
	IBS	308.6	282.10	108.07	1041.08				
	HC	219.3	70.20	130.20	657.15				
Broccoli	MDD	5.29	3.16	1.52	21.85	6.20	0.045	MDD > HC	0,039
	IBS	4.73	2.72	1.79	10.43				
	HC	3.56	1.04	1.62	6.40				
Carrot	MDD	4.23	8.65	1.70	34.00	3.80	0.150	-	-
	IBS	4.67	3.69	1.77	10.69				
	HC	3.55	1.77	1.23	6.53				
Red cabbage	MDD	6.31	6.23	1.77	44.06	4.21	0.122	-	-
	IBS	4.90	4.01	2.50	9.13				
	HC	3.86	1.88	1.61	13.33				
Cucumber	MDD	3.99	4.37	1.76	36.39	3.02	0.220	-	-
	IBS	3.82	4.33	1.97	16.47				
	HC	3.07	1.41	1.31	7.61				
Bell pepper	MDD	3.59	6.05	0.97	20.70	3.23	0.199	-	-
	IBS	4.29	5.48	1.46	23.58				
	HC	2.39	2.38	1.09	11.51				
Tomatoes	MDD	4.38	4.50	1.46	29.47	0.97	0.617	-	-
	IBS	4.45	4.35	1.65	20.40				
	HC	3.94	1.92	1.41	32.64				
Celery	MDD	6.15	6.80	1.58	28.20	8.03	0.017*	MDD > HC	0,019
	IBS	4.53	4.74	1.52	12.43				
	HC	2.87	2.14	1.14	7.32				
Soy beans	MDD	3.89	3.52	1.92	13.91	4.45	0.108	-	-
	IBS	2.59	2.04	2.07	26.33				
	HC	2.73	1.72	1.65	8.01				
Pineapple	MDD	3.82	6.15	0.83	61.53	0.63	0.730	-	-
	IBS	5.02	6.62	0.94	27.67				
	HC	2.55	5.35	1.44	17.26				

Watermelon	MDD	4.21	6.75	1.02	37.09	3.42	0.181	-	-
	IBS	3.06	2.75	0.92	18.68				
	HC	2.10	0.97	0.81	10.73				
Cherry	MDD	5.15	5.57	1.24	48.20	3.49	0.175	-	-
	IBS	4.12	3.48	1.52	21.84				
	HC	3.43	2.53	29.47	18.14				
Horseradish	MDD	4.08	3.66	1.42	48.10	6.98	0.030	MDD > HC	0,024
	IBS	4.02	2.68	1.51	11.43				
	HC	2.77	0.89	1.08	6.79				
Garlic	MDD	6.33	7.02	1.01	75.64	7.88	0.017*	MDD > HC	0,015
	IBS	2.92	4.98	0.75	15.83				
	HC	2.49	1.19	0.95	10.77				
Mustard seeds	MDD	3.38	3.34	1.06	13.39	0.66	0.720	-	-
	IBS	2.73	2.68	1.42	8.76				
	HC	2.64	1.74	1.51	15.27				
Pork	MDD	3.36	1.77	2.57	6.71	1.12	0.572	-	-
	IBS	4.26	3.10	1.95	12.79				
	HC	3.80	1.42	1.77	13.87				
Beef	MDD	2.51	1.14	1.45	34.16	1.21	0.545	-	-
	IBS	2.50	1.47	1.37	7.93				
	HC	2.91	1.59	1.59	7.12				
Chicken	MDD	3.32	2.46	1.99	9.03	0.07	0.968	-	-
	IBS	3.21	3.40	1.43	11.30				
	HC	3.17	3.07	1.87	8.32				
Egg	MDD	22.85	21.82	1.77	139.19	0.35	0.839	-	-
	IBS	22.70	32.47	2.38	134.18				
Gluten	MDD	16.44	16.10	5.26	112.61	10.37	0.005*	MDD > IBS	0,010
	IBS	8.87	10.43	2.26	117.87			MDD > HC	0,025
	HC	11.74	7.40	3.94	43.01				
Barley	MDD	5.87	11.41	1.86	42.43	3.66	0.161	-	-
	IBS	5.50	4.98	1.75	24.81				
	HC	3.87	2.88	1.79	19.52				
Oat	MDD	4.83	5.94	1.33	49.89	1.88	0.392	-	-
	IBS	6.51	10.94	1.42	86.70				
	HC	4.18	3.46	1.35	18.49				

Wheat	MDD	15.26	16.38	4.56	99.42	6.79	0.033	MDD > HC	0,043
	IBS	9.31	8.05	2.28	122.13				
	HC	8.92	5.53	3.28	47.22				
Spelt	MDD	12.91	13.23	2.18	53.56	3.30	0.192	-	-
	IBS	7.78	13.29	2.17	138.87				
	HC	7.24	3.17	1.91	30.91				
Rye	MDD	11.41	8.42	3.73	108.92	7.23	0.026	MDD > HC	0,032
	IBS	7.38	8.14	2.10	28.52				
	HC	5.59	4.94	2.08	38.05				
Poppy seeds	MDD	2.88	4.26	1.18	20.46	2.94	0.230	-	-
	IBS	3.38	2.01	0.63	10.39				
	HC	1.93	1.70	0.83	9.72				
Almonds	MDD	1.97	2.50	0.78	10.20	0.43	0.807	-	-
	IBS	2.46	2.56	0.73	5.11				
	HC	2.80	3.66	0.53	9.77				
Hazelnuts	MDD	3.78	4.23	0.87	29.18	2.49	0.288	-	-
	IBS	2.37	2.78	1.09	9.89				
	HC	1.99	1.39	0.88	14.75				
Peanuts	MDD	5.27	7.44	2.30	96.96	3.00	0.223	-	-
	IBS	5.10	4.42	1.44	14.22				
	HC	4.06	2.78	1.30	60.91				
Pistachio	MDD	2.67	2.52	1.12	80.54	1.12	0.572	-	-
	IBS	3.02	5.87	0.93	95.86				
	HC	2.10	1.93	1.25	6.71				
Flax seeds	MDD	2.93	3.86	1.04	12.25	3.10	0.213	-	-
	IBS	3.86	4.00	0.94	41.65				
	HC	2.34	1.87	0.88	15.03				
Sunflower seeds	MDD	6.22	5.84	1.43	46.15	7.07	0.029	MDD > IBS	0,038
	IBS	3.06	2.94	0.91	68.05				
	HC	3.13	2.45	0.74	61.62				
Cray fish	MDD	3.48	3.84	1.62	23.13	1.96	0.375	-	-
	IBS	2.95	1.86	1.66	8.59				
	HC	3.56	1.47	1.64	10.10				

Cow's milk	MDD	35.41	57.53	2.69	143.30	5.20	0.074	-	-
	IBS	29.63	53.70	1.43	140.91				
	HC	10.05	6.52	1.41	425.90				
Milk products	MDD	27.57	39.82	1.62	110.57	7.59	0.025	MDD > HC	0,019
	IBS	20.00	42.78	1.24	119.30				
	HC	6.86	5.46	0.96	86.24				
Cow's cheese	MDD	8.81	25.55	1.97	200.74	4.86	0.088	-	-
	IBS	6.72	16.70	2.04	58.96				
	HC	4.46	3.85	1.92	179.43				
Goat's milk, cheese	MDD	9.48	15.56	1.22	94.57	2.93	0.231	-	-
	IBS	7.08	11.37	1.48	98.37				
	HC	4.39	2.71	1.30	32.45				
Sheep's milk, cheese	MDD	11.38	19.79	1.75	127.68	4.50	0.106	-	-
	IBS	8.16	13.58	1.60	112.14				
	HC	5.15	4.40	1.72	56.41				
Honey	MDD	4.61	4.07	2.04	29.86	0.76	0.686	-	-
	IBS	4.10	2.33	2.04	17.75				
	HC	4.37	3.48	1.47	13.83				
Bakery yeast	MDD	5.36	3.73	2.53	26.15	0.93	0.628	-	-
	IBS	5.43	5.57	2.41	16.15				
	HC	4.34	4.40	1.92	17.26				

*differences statistically significant also after *post-hoc* analysis
G – group; *M* – median; IQR - interquartile range; H – H-value; p – p-value
