# Supplementary material for: The Food-Specific Serum IgG Reactivity in Major Depressive Disorder Patients, Irritable Bowel Syndrome Patients and Healthy Controls
Source: Nutrients. 2018 Apr 28;10(5):548. doi: 10.3390/nu10050548 (PMC5986428; doi:10.3390/nu10050548)
Supplement: Supplementary file 1 [file nutrients-10-00548-s001.zip › supplementary materials/Table S3.pdf]

Table S3. Frequency of elevated IgG levels in examined groups

IgG	Level	MDD n (%)	IBS n (%)	HC n (%)
Broccoli	not elevated	17 (77.27)	19 (86.36)	21 (100.00)
	elevated	4 (18.18)	3 (13.64)	0 (0.00)
	significantly elevated	1 (4.55)	0(0.00)	0 (0.00)
Carrot	not elevated	14 (63.64)	20 (90.91)	21 (100.00)
	elevated	5 (22.73)	2 (9.09)	0 (0.00)
	significantly elevated	3 (13.64)	0 (0.00)	0 (0.00)
Red cabbage	not elevated	15 (68.18)	19 (86.36)	20 (95.24)
	elevated	4 (18.18)	3 (13.64)	1 (4.76)
	significantly elevated	3 (13.64)	0 (0.00)	0 (0.00)
Cucumber	not elevated	17 (77.27)	18 (81.82)	20 (95.24)
	elevated	4 (18.18)	4 (18.18)	1 (4.76)
	significantly elevated	1 (4.55)	0 (0.00)	0 (0.00)
Bell pepper	not elevated	16 (72.73)	16 (72.73)	18 (85.71)
	elevated	5 (22.73)	5 (22.73)	3 (14.29)
	significantly elevated	1 (4.55)	1 (4.55)	0 (0.00)
Tomatoes	not elevated	18 (81.82)	17 (77.27)	19 (90.48)
	elevated	3 (13.64)	4 (18.18)	1 (4.76)
	significantly elevated	1 (4.55)	1 (4.55)	1 (4.76)
Celery	not elevated	13 (59.09)	16 (72.73)	21 (100.00)
	elevated	6 (27.27)	6 (27.27)	0 (0.00)
	significantly elevated	3 (13.64)	0 (0.00)	0 (0.00)
Soy beans	not elevated	18 (81.82)	19 (86.36)	20 (95.24)
	elevated	4 (18.18)	2 (9.09)	1 (4.76)
	significantly elevated	0 (0.00)	1 (4.55)	0 (0.00)
Pineapple	not elevated	16 (72.73)	15 (68.18)	16 (76.19)
	elevated	5 (22.73)	5 (22.73)	5 (23.81)
	significantly elevated	1 (4.55)	2 (9.09)	0 (0.00)
Watermelon	not elevated	16 (72.73)	19 (86.36)	20 (95.24)
	elevated	4 (18.18)	3 (13.64)	1 (4.76)
	significantly elevated	2 (9.09)	0 (0.00)	0 (0.00)
Cherry	not elevated	15 (68.18)	19 (86.36)	19 (90.48)
	elevated	4 (18.18)	2 (9.09)	2 (9.52)
	significantly elevated	3 (13.64)	1 (4.55)	0 (0.00)
Horseradish	not elevated	17 (77.27)	19 (86.36)	21 (100.00)
	elevated	3 (13.64)	3 (13.64)	0 (0.00)
	significantly elevated	2 (9.09)	0 (0.00)	0 (0.00)

Garlic	not elevated	14 (63.64)	17 (77.27)	19 (90.48)
	elevated	5 (22.73)	5 (22.73)	2 (9.52)
	significantly elevated	3 (13.64)	0 (0.00)	0 (0.00)
Mustard seeds	not elevated	20 (90.91)	21 (95.45)	20 (95.24)
	elevated	2 (9.09)	1 (4.55)	1 (4.76)
	significantly elevated	0 (0.00)	0 (0.00)	0 (0.00)
Pork	not elevated	22 (100.00)	20 (90.91)	19 (90.48)
	elevated	0 (0.00)	2 (9.09)	2 (9.52)
	significantly elevated	0 (0.00)	0 (0.00)	0 (0.00)
Beef	not elevated	21 (95.45)	21 (95.45)	21 (100.00)
	elevated	0 (0.00)	1 (4.55)	0 (0.00)
	significantly elevated	1 (4.55)	0 (0.00)	0 (0.00)
Chicken	not elevated	19 (86.36)	20 (90.91)	19 (90.48)
	elevated	3 (13.64)	2 (9.09)	2 (9.52)
	significantly elevated	0 (0.00)	0 (0.00)	0 (0.00)
Egg	not elevated	5 (22.73)	4 (18.18)	3 (14.29)
	elevated	5 (22.73)	6 (27.27)	7 (33.33)
	significantly elevated	12 (54.55)	12 (54.55)	11 (52.38)
Gluten	not elevated	2 (9.09)	8 (36.36)	7 (33.33)
	elevated	11 (50.00)	10 (45.45)	11 (52.38)
	significantly elevated	9 (40.91)	4 (18.18)	3 (14.29)
Barley	not elevated	14 (63.64)	15 (68.18)	16 (76.19)
	elevated	4 (18.18)	6 (27.27)	5 (23.81)
	significantly elevated	4 (18.18)	1 (4.55)	0 (0.00)
Oat	not elevated	15 (68.18)	14 (63.64)	17 (80.95)
	elevated	5 (22.73)	6 (27.27)	4 (19.05)
	significantly elevated	2 (9.09)	2 (9.09)	0 (0.00)
Wheat	not elevated	6 (27.27)	10 (45.45)	7 (33.33)
	elevated	9 (40.91)	9 (40.91)	13 (61.90)
	significantly elevated	7 (31.82)	3 (13.64)	1 (4.76)
Spelt	not elevated	8 (36.36)	10 (45.45)	13 (61.90)
	elevated	9 (40.91)	9 (40.91)	7 (33.33)
	significantly elevated	5 (22.73)	3 (13.64)	1 (4.76)
Rye	not elevated	7 (31.82)	11 (50.00)	15 (71.43)
	elevated	12 (54.55)	9 (40.91)	4 (19.05)
	significantly elevated	3 (13.64)	2 (9.09)	2 (9.52)
Poppy seeds	not elevated	17 (77.27)	20 (90.91)	20 (95.24)
	elevated	4 (18.18)	2 (9.09)	1 (4.76)
	significantly elevated	1 (4.55)	0 (0.00)	0 (0.00)
Almonds	not elevated	19 (86.36)	22 (100.00)	19 (90.48)
	elevated	3 (13.64)	0 (0.00)	2 (9.52)
	significantly elevated	0 (0.00)	0 (0.00)	0 (0.00)

Hazelnuts	not elevated	18 (81.82)	21 (95.45)	19 (90.48)
	elevated	3 (13.64)	1 (4.55)	2 (9.52)
	significantly elevated	1 (4.55)	0 (0.00)	0 (0.00)
Peanuts	not elevated	15 (68.18)	18 (81.82)	17 (80.95)
	elevated	5 (22.73)	4 (18.18)	3 (14.29)
	significantly elevated	2 (9.09)	0 (0.00)	1 (4.76)
Pistachio	not elevated	20 (90.91)	17 (77.27)	21 (100.00)
	elevated	1 (4.55)	4 (18.18)	0 (0.00)
	significantly elevated	1 (4.55)	1 (4.55)	0 (0.00)
Flax seeds	not elevated	18 (81.82)	17 (77.27)	20 (95.24)
	elevated	4 (18.18)	2 (9.09)	1 (4.76)
	significantly elevated	0 (0.00)	3 (13.64)	0 (0.00)
Sunflower seeds	not elevated	13 (59.09)	20 (90.91)	19 (90.48)
	elevated	8 (36.36)	1 (4.55)	1 (4.76)
	significantly elevated	1 (4.55)	1 (4.55)	1 (4.76)
Cray fish	not elevated	19 (86.36)	20 (90.91)	19 (90.48)
	elevated	2 (9.09)	2 (9.09)	2 (9.52)
	significantly elevated	1 (4.55)	0 (0.00)	0 (0.00)
Cow’s milk	not elevated	3 (13.64)	8 (36.36)	5 (23.81)
	elevated	7 (31.82)	2 (9.09)	12 (57.14)
	significantly elevated	12 (54.55)	12 (54.55)	4 (19.05)
Milk products	not elevated	5 (22.73)	9 (40.91)	12 (57.14)
	elevated	4 (18.18)	2 (9.09)	7 (33.33)
	significantly elevated	13 (59.09)	11 (50.00)	2 (9.52)
Cow’s cheese	not elevated	8 (36.36)	13 (59.09)	15 (71.43)
	elevated	7 (31.82)	3 (13.64)	5 (23.81)
	significantly elevated	7 (31.82)	6 (27.27)	1 (4.76)
Goat’s milk, cheese	not elevated	9 (40.91)	11 (50.00)	18 (85.71)
	elevated	9 (40.91)	6 (27.27)	1 (4.76)
	significantly elevated	4 (18.18)	5 (22.73)	2 (9.52)
Sheep’s milk, cheese	not elevated	9 (40.91)	11 (50.00)	17 (80.95)
	elevated	5 (22.73)	7 (31.82)	2 (9.52)
	significantly elevated	8 (36.36)	4 (18.18)	2 (9.52)
Honey	not elevated	17 (77.27)	19 (86.36)	16 (76.19)
	elevated	3 (13.64)	3 (13.64)	5 (23.81)
	significantly elevated	2 (9.09)	0 (0.00)	0 (0.00)
Bakery yeast	not elevated	16 (72.73)	15 (68.18)	15 (71.43)
	elevated	4 (18.18)	7 (31.82)	6 (28.57)
	significantly elevated	2 (9.09)	0 (0.00)	0 (0.00)

interpretation according to manufacturer's descriptions in which values were considered as: not elevated for tirtes below 7.5 μg/ml, elevated for tirtes 7.5 – 20.0 μg/ml, significantly elevated for tirtes above 20.0 μg/ml
