# Supplementary material for: The Food-Specific Serum IgG Reactivity in Major Depressive Disorder Patients, Irritable Bowel Syndrome Patients and Healthy Controls
Source: Nutrients. 2018 Apr 28;10(5):548. doi: 10.3390/nu10050548 (PMC5986428; doi:10.3390/nu10050548)
Supplement: Supplementary file 1 [file nutrients-10-00548-s001.zip › supplementary materials/Table S2.doc]

**Table S2.** Differences in serum IgG levels against tested food proteins

| **IgG** | **G** | ***M*** | **IQR** | **Min** | **Max** | **Kruskal-Wallis H Test for 3 groups** | | ***Post-hoc* analysis** | |
| --- | --- | --- | --- | --- | --- | --- | --- | --- | --- |
|  |  |  |  |  |  | **H** | **p** | **groups** | **p** |
| Total  IgG | MDD | 403.9 | 365.10 | 109.68 | 1075.11 | 7.90 | 0.019 | MDD > HC | 0,004 |
|  | IBS | 308.6 | 282.10 | 108.07 | 1041.08 |  |  |  |  |
|  | HC | 219.3 | 70.20 | 130.20 | 657.15 |  |  |  |  |
| Broccoli | MDD | 5.29 | 3.16 | 1.52 | 21.85 | 6.20 | 0.045 | MDD > HC | 0,039 |
|  | IBS | 4.73 | 2.72 | 1.79 | 10.43 |  |  |  |  |
|  | HC | 3.56 | 1.04 | 1.62 | 6.40 |  |  |  |  |
| Carrot | MDD | 4.23 | 8.65 | 1.70 | 34.00 | 3.80 | 0.150 | - | - |
|  | IBS | 4.67 | 3.69 | 1.77 | 10.69 |  |  |  |  |
|  | HC | 3.55 | 1.77 | 1.23 | 6.53 |  |  |  |  |
| Red cabbage | MDD | 6.31 | 6.23 | 1.77 | 44.06 | 4.21 | 0.122 | - | - |
|  | IBS | 4.90 | 4.01 | 2.50 | 9.13 |  |  |  |  |
|  | HC | 3.86 | 1.88 | 1.61 | 13.33 |  |  |  |  |
| Cucumber | MDD | 3.99 | 4.37 | 1.76 | 36.39 | 3.02 | 0.220 | - | - |
|  | IBS | 3.82 | 4.33 | 1.97 | 16.47 |  |  |  |  |
|  | HC | 3.07 | 1.41 | 1.31 | 7.61 |  |  |  |  |
| Bell pepper | MDD | 3.59 | 6.05 | 0.97 | 20.70 | 3.23 | 0.199 | - | - |
|  | IBS | 4.29 | 5.48 | 1.46 | 23.58 |  |  |  |  |
|  | HC | 2.39 | 2.38 | 1.09 | 11.51 |  |  |  |  |
| Tomatoes | MDD | 4.38 | 4.50 | 1.46 | 29.47 | 0.97 | 0.617 | - | - |
|  | IBS | 4.45 | 4.35 | 1.65 | 20.40 |  |  |  |  |
|  | HC | 3.94 | 1.92 | 1.41 | 32.64 |  |  |  |  |
| Celery | MDD | 6.15 | 6.80 | 1.58 | 28.20 | 8.03 | 0.017* | MDD > HC | 0,019 |
|  | IBS | 4.53 | 4.74 | 1.52 | 12.43 |  |  |  |  |
|  | HC | 2.87 | 2.14 | 1.14 | 7.32 |  |  |  |  |
| Soy beans | MDD | 3.89 | 3.52 | 1.92 | 13.91 | 4.45 | 0.108 | - | - |
|  | IBS | 2.59 | 2.04 | 2.07 | 26.33 |  |  |  |  |
|  | HC | 2.73 | 1.72 | 1.65 | 8.01 |  |  |  |  |
| Pineapple | MDD | 3.82 | 6.15 | 0.83 | 61.53 | 0.63 | 0.730 | - | - |
|  | IBS | 5.02 | 6.62 | 0.94 | 27.67 |  |  |  |  |
|  | HC | 2.55 | 5.35 | 1.44 | 17.26 |  |  |  |  |
| Watermelon | MDD | 4.21 | 6.75 | 1.02 | 37.09 | 3.42 | 0.181 | - | - |
|  | IBS | 3.06 | 2.75 | 0.92 | 18.68 |  |  |  |  |
|  | HC | 2.10 | 0.97 | 0.81 | 10.73 |  |  |  |  |
| Cherry | MDD | 5.15 | 5.57 | 1.24 | 48.20 | 3.49 | 0.175 | - | - |
|  | IBS | 4.12 | 3.48 | 1.52 | 21.84 |  |  |  |  |
|  | HC | 3.43 | 2.53 | 29.47 | 18.14 |  |  |  |  |
| Horseradish | MDD | 4.08 | 3.66 | 1.42 | 48.10 | 6.98 | 0.030 | MDD > HC | 0,024 |
|  | IBS | 4.02 | 2.68 | 1.51 | 11.43 |  |  |  |  |
|  | HC | 2.77 | 0.89 | 1.08 | 6.79 |  |  |  |  |
| Garlic | MDD | 6.33 | 7.02 | 1.01 | 75.64 | 7.88 | 0.017* | MDD > HC | 0,015 |
|  | IBS | 2.92 | 4.98 | 0.75 | 15.83 |  |  |  |  |
|  | HC | 2.49 | 1.19 | 0.95 | 10.77 |  |  |  |  |
| Mustard seeds | MDD | 3.38 | 3.34 | 1.06 | 13.39 | 0.66 | 0.720 | - | - |
|  | IBS | 2.73 | 2.68 | 1.42 | 8.76 |  |  |  |  |
|  | HC | 2.64 | 1.74 | 1.51 | 15.27 |  |  |  |  |
| Pork | MDD | 3.36 | 1.77 | 2.57 | 6.71 | 1.12 | 0.572 | - | - |
|  | IBS | 4.26 | 3.10 | 1.95 | 12.79 |  |  |  |  |
|  | HC | 3.80 | 1.42 | 1.77 | 13.87 |  |  |  |  |
| Beef | MDD | 2.51 | 1.14 | 1.45 | 34.16 | 1.21 | 0.545 | - | - |
|  | IBS | 2.50 | 1.47 | 1.37 | 7.93 |  |  |  |  |
|  | HC | 2.91 | 1.59 | 1.59 | 7.12 |  |  |  |  |
| Chicken | MDD | 3.32 | 2.46 | 1.99 | 9.03 | 0.07 | 0.968 | - | - |
|  | IBS | 3.21 | 3.40 | 1.43 | 11.30 |  |  |  |  |
|  | HC | 3.17 | 3.07 | 1.87 | 8.32 |  |  |  |  |
| Egg | MDD | 22.85 | 21.82 | 1.77 | 139.19 | 0.35 | 0.839 | - | - |
|  | IBS | 22.70 | 32.47 | 2.38 | 134.18 |  |  |  |  |
| Gluten | MDD | 16.44 | 16.10 | 5.26 | 112.61 | 10.37 | 0.005* | MDD > IBS  MDD > HC | 0,010  0,025 |
|  | IBS | 8.87 | 10.43 | 2.26 | 117.87 |  |  |  |  |
|  | HC | 11.74 | 7.40 | 3.94 | 43.01 |  |  |  |  |
| Barley | MDD | 5.87 | 11.41 | 1.86 | 42.43 | 3.66 | 0.161 | - | - |
|  | IBS | 5.50 | 4.98 | 1.75 | 24.81 |  |  |  |  |
|  | HC | 3.87 | 2.88 | 1.79 | 19.52 |  |  |  |  |
| Oat | MDD | 4.83 | 5.94 | 1.33 | 49.89 | 1.88 | 0.392 | - | - |
|  | IBS | 6.51 | 10.94 | 1.42 | 86.70 |  |  |  |  |
|  | HC | 4.18 | 3.46 | 1.35 | 18.49 |  |  |  |  |
| Wheat | MDD | 15.26 | 16.38 | 4.56 | 99.42 | 6.79 | 0.033 | MDD > HC | 0,043 |
|  | IBS | 9.31 | 8.05 | 2.28 | 122.13 |  |  |  |  |
|  | HC | 8.92 | 5.53 | 3.28 | 47.22 |  |  |  |  |
| Spelt | MDD | 12.91 | 13.23 | 2.18 | 53.56 | 3.30 | 0.192 | - | - |
|  | IBS | 7.78 | 13.29 | 2.17 | 138.87 |  |  |  |  |
|  | HC | 7.24 | 3.17 | 1.91 | 30.91 |  |  |  |  |
| Rye | MDD | 11.41 | 8.42 | 3.73 | 108.92 | 7.23 | 0.026 | MDD > HC | 0,032 |
|  | IBS | 7.38 | 8.14 | 2.10 | 28.52 |  |  |  |  |
|  | HC | 5.59 | 4.94 | 2.08 | 38.05 |  |  |  |  |
| Poppy seeds | MDD | 2.88 | 4.26 | 1.18 | 20.46 | 2.94 | 0.230 | - | - |
|  | IBS | 3.38 | 2.01 | 0.63 | 10.39 |  |  |  |  |
|  | HC | 1.93 | 1.70 | 0.83 | 9.72 |  |  |  |  |
| Almonds | MDD | 1.97 | 2.50 | 0.78 | 10.20 | 0.43 | 0.807 | - | - |
|  | IBS | 2.46 | 2.56 | 0.73 | 5.11 |  |  |  |  |
|  | HC | 2.80 | 3.66 | 0.53 | 9.77 |  |  |  |  |
| Hazelnuts | MDD | 3.78 | 4.23 | 0.87 | 29.18 | 2.49 | 0.288 | - | - |
|  | IBS | 2.37 | 2.78 | 1.09 | 9.89 |  |  |  |  |
|  | HC | 1.99 | 1.39 | 0.88 | 14.75 |  |  |  |  |
| Peanuts | MDD | 5.27 | 7.44 | 2.30 | 96.96 | 3.00 | 0.223 | - | - |
|  | IBS | 5.10 | 4.42 | 1.44 | 14.22 |  |  |  |  |
|  | HC | 4.06 | 2.78 | 1.30 | 60.91 |  |  |  |  |
| Pistachio | MDD | 2.67 | 2.52 | 1.12 | 80.54 | 1.12 | 0.572 | - | - |
|  | IBS | 3.02 | 5.87 | 0.93 | 95.86 |  |  |  |  |
|  | HC | 2.10 | 1.93 | 1.25 | 6.71 |  |  |  |  |
| Flax seeds | MDD | 2.93 | 3.86 | 1.04 | 12.25 | 3.10 | 0.213 | - | - |
|  | IBS | 3.86 | 4.00 | 0.94 | 41.65 |  |  |  |  |
|  | HC | 2.34 | 1.87 | 0.88 | 15.03 |  |  |  |  |
| Sunflower seeds | MDD | 6.22 | 5.84 | 1.43 | 46.15 | 7.07 | 0.029 | MDD > IBS | 0,038 |
|  | IBS | 3.06 | 2.94 | 0.91 | 68.05 |  |  |  |  |
|  | HC | 3.13 | 2.45 | 0.74 | 61.62 |  |  |  |  |
| Cray fish | MDD | 3.48 | 3.84 | 1.62 | 23.13 | 1.96 | 0.375 | - | - |
|  | IBS | 2.95 | 1.86 | 1.66 | 8.59 |  |  |  |  |
|  | HC | 3.56 | 1.47 | 1.64 | 10.10 |  |  |  |  |

| Cow’s milk | MDD | 35.41 | 57.53 | 2.69 | 143.30 | 5.20 | 0.074 | - | - |
| --- | --- | --- | --- | --- | --- | --- | --- | --- | --- |
|  | IBS | 29.63 | 53.70 | 1.43 | 140.91 |  |  |  |  |
|  | HC | 10.05 | 6.52 | 1.41 | 425.90 |  |  |  |  |
| Milk products | MDD | 27.57 | 39.82 | 1.62 | 110.57 | 7.59 | 0.025 | MDD > HC | 0,019 |
|  | IBS | 20.00 | 42.78 | 1.24 | 119.30 |  |  |  |  |
|  | HC | 6.86 | 5.46 | 0.96 | 86.24 |  |  |  |  |
| Cow’s cheese | MDD | 8.81 | 25.55 | 1.97 | 200.74 | 4.86 | 0.088 | - | - |
|  | IBS | 6.72 | 16.70 | 2.04 | 58.96 |  |  |  |  |
|  | HC | 4.46 | 3.85 | 1.92 | 179.43 |  |  |  |  |
| Goat’s milk, cheese | MDD | 9.48 | 15.56 | 1.22 | 94.57 | 2.93 | 0.231 | - | - |
|  | IBS | 7.08 | 11.37 | 1.48 | 98.37 |  |  |  |  |
|  | HC | 4.39 | 2.71 | 1.30 | 32.45 |  |  |  |  |
| Sheep’s milk, cheese | MDD | 11.38 | 19.79 | 1.75 | 127.68 | 4.50 | 0.106 | - | - |
|  | IBS | 8.16 | 13.58 | 1.60 | 112.14 |  |  |  |  |
|  | HC | 5.15 | 4.40 | 1.72 | 56.41 |  |  |  |  |
| Honey | MDD | 4.61 | 4.07 | 2.04 | 29.86 | 0.76 | 0.686 | - | - |
|  | IBS | 4.10 | 2.33 | 2.04 | 17.75 |  |  |  |  |
|  | HC | 4.37 | 3.48 | 1.47 | 13.83 |  |  |  |  |
| Bakery yeast | MDD | 5.36 | 3.73 | 2.53 | 26.15 | 0.93 | 0.628 | - | - |
|  | IBS | 5.43 | 5.57 | 2.41 | 16.15 |  |  |  |  |
|  | HC | 4.34 | 4.40 | 1.92 | 17.26 |  |  |  |  |

*differences statistically significant also after *post-hoc* analysis
G – group; *M* – median; IQR - interquartile range; H – H-value; p – p-value
