# Supplementary material for: The Food-Specific Serum IgG Reactivity in Major Depressive Disorder Patients, Irritable Bowel Syndrome Patients and Healthy Controls
Source: Nutrients. 2018 Apr 28;10(5):548. doi: 10.3390/nu10050548 (PMC5986428; doi:10.3390/nu10050548)
Supplement: Supplementary file 1 [file nutrients-10-00548-s001.zip › supplementary materials/Table S3.doc]

**Table S3.** Frequency of elevated IgG levels in examined groups

| **IgG** | **Level** | **MDD**  **n (%)** | **IBS**  **n (%)** | **HC**  **n (%)** |
| --- | --- | --- | --- | --- |
| Broccoli | not elevated | 17 (77.27) | 19 (86.36) | 21 (100.00) |
|  | elevated | 4 (18.18) | 3 (13.64) | 0 (0.00) |
|  | significantly elevated | 1 (4.55) | 0( 0.00) | 0 (0.00) |
| Carrot | not elevated | 14 (63.64) | 20 (90.91) | 21 (100.00) |
|  | elevated | 5 (22.73) | 2 (9.09) | 0 (0.00) |
|  | significantly elevated | 3 (13.64) | 0 (0.00) | 0 (0.00) |
| Red cabbage | not elevated | 15 (68.18) | 19 (86.36) | 20 (95.24) |
|  | elevated | 4 (18.18) | 3 (13.64) | 1 (4.76) |
|  | significantly elevated | 3 (13.64) | 0 (0.00) | 0 (0.00) |
| Cucumber | not elevated | 17 (77.27) | 18 (81.82) | 20 (95.24) |
|  | elevated | 4 (18.18) | 4 (18.18) | 1 (4.76) |
|  | significantly elevated | 1 (4.55) | 0 (0.00) | 0 (0.00) |
| Bell pepper | not elevated | 16 (72.73) | 16 (72.73) | 18 (85.71) |
|  | elevated | 5 (22.73) | 5 (22.73) | 3 (14.29) |
|  | significantly elevated | 1 (4.55) | 1 (4.55) | 0 (0.00) |
| Tomatoes | not elevated | 18 (81.82) | 17 (77.27) | 19 (90.48) |
|  | elevated | 3 (13.64) | 4 (18.18) | 1 (4.76) |
|  | significantly elevated | 1 (4.55) | 1 (4.55) | 1 (4.76) |
| Celery | not elevated | 13 (59.09) | 16 (72.73) | 21 (100.00) |
|  | elevated | 6 (27.27) | 6 (27.27) | 0 (0.00) |
|  | significantly elevated | 3 (13.64) | 0 (0.00) | 0 (0.00) |
| Soy beans | not elevated | 18 (81.82) | 19 (86.36) | 20 (95.24) |
|  | elevated | 4 (18.18) | 2 (9.09) | 1 (4.76) |
|  | significantly elevated | 0 (0.00) | 1 (4.55) | 0 (0.00) |
| Pineapple | not elevated | 16 (72.73) | 15 (68.18) | 16 (76.19) |
|  | elevated | 5 (22.73) | 5 (22.73) | 5 (23.81) |
|  | significantly elevated | 1 (4.55) | 2 (9.09) | 0 ( 0.00) |
| Watermelon | not elevated | 16 (72.73) | 19 (86.36) | 20 (95.24) |
|  | elevated | 4 (18.18) | 3 (13.64) | 1 (4.76) |
|  | significantly elevated | 2 (9.09) | 0 (0.00) | 0 (0.00) |
| Cherry | not elevated | 15 (68.18) | 19 (86.36) | 19 (90.48) |
|  | elevated | 4 (18.18) | 2 (9.09) | 2 (9.52) |
|  | significantly elevated | 3 (13.64) | 1 (4.55) | 0 (0.00) |
| Horseradish | not elevated | 17 (77.27) | 19 (86.36) | 21 (100.00) |
|  | elevated | 3 (13.64) | 3 (13.64) | 0 (0.00) |
|  | significantly elevated | 2 (9.09) | 0 (0.00) | 0 (0.00) |
| Garlic | not elevated | 14 (63.64) | 17 (77.27) | 19 (90.48) |
|  | elevated | 5 (22.73) | 5 (22.73) | 2 (9.52) |
|  | significantly elevated | 3 (13.64) | 0 (0.00) | 0 (0.00) |
| Mustard seeds | not elevated | 20 (90.91) | 21 (95.45) | 20 (95.24) |
|  | elevated | 2 (9.09) | 1 (4.55) | 1 (4.76) |
|  | significantly elevated | 0 (0.00) | 0 (0.00) | 0 (0.00) |
| Pork | not elevated | 22 (100.00) | 20 (90.91) | 19 (90.48) |
|  | elevated | 0 (0.00) | 2 (9.09) | 2 (9.52) |
|  | significantly elevated | 0 (0.00) | 0 (0.00) | 0 (0.00) |
| Beef | not elevated | 21 (95.45) | 21 (95.45) | 21 (100.00) |
|  | elevated | 0 (0.00) | 1 (4.55) | 0 (0.00) |
|  | significantly elevated | 1 (4.55) | 0 (0.00) | 0 (0.00) |
| Chicken | not elevated | 19 (86.36) | 20 (90.91) | 19 (90.48) |
|  | elevated | 3 (13.64) | 2 (9.09) | 2 (9.52) |
|  | significantly elevated | 0 (0.00) | 0 (0.00) | 0 (0.00) |
| Egg | not elevated | 5 (22.73) | 4 (18.18) | 3 (14.29) |
|  | elevated | 5 (22.73) | 6 (27.27) | 7 (33.33) |
|  | significantly elevated | 12 (54.55) | 12 (54.55) | 11 (52.38) |
| Gluten | not elevated | 2 (9.09) | 8 (36.36) | 7 (33.33) |
|  | elevated | 11 (50.00) | 10 (45.45) | 11 (52.38) |
|  | significantly elevated | 9 (40.91) | 4 (18.18) | 3 (14.29) |
| Barley | not elevated | 14 (63.64) | 15 (68.18) | 16 (76.19) |
|  | elevated | 4 (18.18) | 6 (27.27) | 5 (23.81) |
|  | significantly elevated | 4 (18.18) | 1 (4.55) | 0 (0.00) |
| Oat | not elevated | 15 (68.18) | 14 (63.64) | 17 (80.95) |
|  | elevated | 5 (22.73) | 6 (27.27) | 4 (19.05) |
|  | significantly elevated | 2 (9.09) | 2 (9.09) | 0 (0.00) |
| Wheat | not elevated | 6 (27.27) | 10 (45.45) | 7 (33.33) |
|  | elevated | 9 (40.91) | 9 (40.91) | 13 (61.90) |
|  | significantly elevated | 7 (31.82) | 3 (13.64) | 1 (4.76) |
| Spelt | not elevated | 8 (36.36) | 10 (45.45) | 13 (61.90) |
|  | elevated | 9 (40.91) | 9 (40.91) | 7 (33.33) |
|  | significantly elevated | 5 (22.73) | 3 (13.64) | 1 (4.76) |
| Rye | not elevated | 7 (31.82) | 11 (50.00) | 15 (71.43) |
|  | elevated | 12 (54.55) | 9 (40.91) | 4 (19.05) |
|  | significantly elevated | 3 (13.64) | 2 (9.09) | 2 (9.52) |
| Poppy seeds | not elevated | 17 (77.27) | 20 (90.91) | 20 (95.24) |
|  | elevated | 4 (18.18) | 2 (9.09) | 1 (4.76) |
|  | significantly elevated | 1 (4.55) | 0 (0.00) | 0 (0.00) |
| Almonds | not elevated | 19 (86.36) | 22 (100.00) | 19 (90.48) |
|  | elevated | 3 (13.64) | 0 (0.00) | 2 (9.52) |
|  | significantly elevated | 0 (0.00) | 0 (0.00) | 0 (0.00) |
| Hazelnuts | not elevated | 18 (81.82) | 21 (95.45) | 19 (90.48) |
|  | elevated | 3 (13.64) | 1 (4.55) | 2 (9.52) |
|  | significantly elevated | 1 (4.55) | 0 (0.00) | 0 (0.00) |
| Peanuts | not elevated | 15 (68.18) | 18 (81.82) | 17 (80.95) |
|  | elevated | 5 (22.73) | 4 (18.18) | 3 (14.29) |
|  | significantly elevated | 2 (9.09) | 0 (0.00) | 1 (4.76) |
| Pistachio | not elevated | 20 (90.91) | 17 (77.27) | 21 (100.00) |
|  | elevated | 1 (4.55) | 4 (18.18) | 0 (0.00) |
|  | significantly elevated | 1 (4.55) | 1 (4.55) | 0 (0.00) |
| Flax seeds | not elevated | 18 (81.82) | 17 (77.27) | 20 (95.24) |
|  | elevated | 4 (18.18) | 2 (9.09) | 1 (4.76) |
|  | significantly elevated | 0 (0.00) | 3 (13.64) | 0 (0.00) |
| Sunflower seeds | not elevated | 13 (59.09) | 20 (90.91) | 19 (90.48) |
|  | elevated | 8 (36.36) | 1 (4.55) | 1 (4.76) |
|  | significantly elevated | 1 (4.55) | 1 (4.55) | 1 (4.76) |
| Cray fish | not elevated | 19 (86.36) | 20 (90.91) | 19 (90.48) |
|  | elevated | 2 (9.09) | 2 (9.09) | 2 (9.52) |
|  | significantly elevated | 1 (4.55) | 0 (0.00) | 0 (0.00) |
| Cow’s milk | not elevated | 3 (13.64) | 8 (36.36) | 5 (23.81) |
|  | elevated | 7 (31.82) | 2 (9.09) | 12 (57.14) |
|  | significantly elevated | 12 (54.55) | 12 (54.55) | 4 (19.05) |
| Milk products | not elevated | 5 (22.73) | 9 (40.91) | 12 (57.14) |
|  | elevated | 4 (18.18) | 2 (9.09) | 7 (33.33) |
|  | significantly elevated | 13 (59.09) | 11 (50.00) | 2 (9.52) |
| Cow’s cheese | not elevated | 8 (36.36) | 13 (59.09) | 15 (71.43) |
|  | elevated | 7 (31.82) | 3 (13.64) | 5 (23.81) |
|  | significantly elevated | 7 (31.82) | 6 (27.27) | 1 (4.76) |
| Goat’s milk, cheese | not elevated | 9 (40.91) | 11 (50.00) | 18 (85.71) |
|  | elevated | 9 (40.91) | 6 (27.27) | 1 (4.76) |
|  | significantly elevated | 4 (18.18) | 5 (22.73) | 2 (9.52) |
| Sheep’s milk, cheese | not elevated | 9 (40.91) | 11 (50.00) | 17 (80.95) |
|  | elevated | 5 (22.73) | 7 (31.82) | 2 (9.52) |
|  | significantly elevated | 8 (36.36) | 4 (18.18) | 2 (9.52) |
| Honey | not elevated | 17 (77.27) | 19 (86.36) | 16 (76.19) |
|  | elevated | 3 (13.64) | 3 (13.64) | 5 (23.81) |
|  | significantly elevated | 2 (9.09) | 0 (0.00) | 0 (0.00) |
| Bakery yeast | not elevated | 16 (72.73) | 15 (68.18) | 15 (71.43) |
|  | elevated | 4 (18.18) | 7 (31.82) | 6 (28.57) |
|  | significantly elevated | 2 (9.09) | 0 (0.00) | 0 (0.00) |

interpretation according to manufacturer’s descriptions in which values were considered as: not elevated for tirtes below 7.5 μg/ml, elevated for tirtes 7.5 – 20.0 μg/ml, significantly elevated for tirtes above 20.0 μg/ml
